# Supplementary material for: WordCommentsAnalyzer: A windows software tool for qualitative research
Source: F1000Res. 2018 Sep 4;7:536. Originally published 2018 May 3. [Version 2] doi: 10.12688/f1000research.14819.2 (PMC6137411; doi:10.12688/f1000research.14819.2)
Supplement: Supplementary file 3 [file f1000research-7-17614-s0002.tgz › b2a79496-4ada-47c4-9682-a99ce867836d.zip!/Eight Iranian Health Journals/Journal of Knowledge and Health_26.docx]

© Journal of Knowledge and Health. This work is licensed under CC BY 4.0 (https://creativecommons.org/licenses/by/4.0/).

Volume & Issue: دوره 6، شماره 4: 1390

Title: بررسي ميزان تأثير افزودن دوز پايين داکليزوماب به پروتکل استاندارد در کاهش پس زدن پيوند در بيماران گيرنده کليه

http://knh.shmu.ac.ir/index.php/site/article/view/118

مقدمه: پيوند کليه، درماني انتخابي براي نارسايي مزمن پيشرفته کليه است. يکي از مهم‌ترين مشکلات درماني بيماران، پيشگيري از رد حاد پيوند مي‌باشد. اين مطالعه با هدف بررسي تأثير دوز کم داکليزوماب در کاهش رد پيوند گيرندگان کليه از دهنده زنده انجام‌شده است. مواد و روش‌ها: اين مطالعه کارآزمايي باليني، در بيمارستان افضلي‌پور کرمان بر روي 120 بيمار گيرنده کليه از دهنده زنده انجام شد. بيماران به‌صورت تصادفي در 2 گروه 60 نفري قرار گرفتند. گروه شاهد داروهاي سيکلوسپورين، مايکوفنولات و پردنيزولون گرفتند. در گروه مداخله داکليزوماب (mg/kg 1) قبل از پيوند و سپس 2 هفته پس از آن نيز به رژيم فوق افزوده شد. وضعيت بيماران حداقل درطول 6 ماه، ازنظر رد پيوند، زمان آن و ميزان عوارض، پيگيري شد. نتایج: ميزان رد حاد پيوند به‌طور مشخصي در گروه مداخله کمتر از شاهد بود (7/6% در مقابل 3/18%، 048/0P=). بقاي 6 ماهه در گروه مداخله 95% و در گروه شاهد 85% بود (029/0P=). تفاوت معناداري در ميزان بقاي 6 ماهه پيوند در زنان گروه مداخله در مقايسه با گروه شاهد مشاهده شد (97% در مقابل 74%، 02/0P=)، ولي در مردان تفاوت معناداري وجود نداشت (94% در مقابل 92%). ميزان بروز عفونت در 2 گروه يکسان بود. نتیجه‌گیری: درمان القايي با دو دوز داکليزوماب در گيرندگان کليه پيوندي از فرد زنده، بروز رد پيوند حاد را کاهش، و بقاي بيمار را به‌خصوص در زنان افزايش مي‌دهد و منجر به افزايش عفونت نمي‌شود.

Title: اثر اسپلنکتومی بر صرع ناشی از پنتیلن تترازول در موش صحرایی نژاد ویستار

http://knh.shmu.ac.ir/index.php/site/article/view/122

مقدمه: باتوجه به اثرات متقابل مغز و طحال، در این پژوهش اثر اسپلنکتومی بر صرع ناشی از پنتیلن تترازول در موش صحرایی نژاد ویستار مورد بررسي قرار گرفته است. مواد و روش‌ها: در این پژوهش از 48 سر موش‌ صحرایی نر و ماده نژاد ویستار، در 3 گروه استفاده شد. گروه‌های آزمایشی برای هریک از دو جنس شامل شاهد منفی‌، شاهد مثبت‌‌ و اسپلنکتومی بود. گروه شاهد منفی تحت عمل جراحی قرار نگرفتند. در گروه شاهد مثبت برش جراحی ایجاد گردید، ولی طحال خارج نشد. در گروه اسپلنکتومی طحال خارج شد. پس از 7 روز، پنتیلن تترازول تزریق و زمان شروع مراحل مختلف تشنجي برحسب ثانيه و عمق حملات‌ تشنجی در 3 گروه به تفكيك جنس، باهم با استفاده از آزمون آناليز واريانس يك‌طرفه مقايسه شد. نتایج: زمان شروع مرحله‌ دوم تشنج در گروه اسپلنکتومی موش‌های نر در مقایسه با گروه‌های شاهد، کاهش معناداری نشان داد (05/0P<). زمان شروع مرحله چهارم تشنجی در گروه اسپلنکتومی از موش‌های ماده نسبت به‌ گروه شاهد منفی مربوطبه این موش‌ها، کاهش معناداری داشت. همچنين ازلحاظ امتیاز تشنج در 2 گروه موش‌هاي نر و ماده، در هر 3 گروه تفاوت معناداري مشاهده نشد. نتیجه‌گیری: اسپلنکتومی موجب تشدید حملات تشنجی ناشی از پنتیلن تترازول در موش صحرایی نژاد ویستار می‌شود. صرع، پنتیلن تترازول، اسپلنکتومی، موش صحرایی.

Title: مقایسه تأثير تمرين مقاومتي بر ميزان گیرنده‌های استیل کولین نیکوتینی (nAChRs) در عضلات كند و تند موش نر ويستار

http://knh.shmu.ac.ir/index.php/site/article/view/124

مقدمه: گیرنده‌های استیل‌کولین نیکوتینی (nAChR)، پروتئین‌های سراسری غشایی و اعضای اصلی سرگروه کانال یونی دریچه لیگاندی هستند كه عمل انتقال سیناپسی را در اتصالات عصبی- ‌عضلانی (NMJ) تعدیل می‌کنند. هدف از تحقیق حاضر، مقایسه تأثير تمرین مقاومتی بر میزان nAChR در عضله نعلي (کند انقباض) و درشت‌نئی قدامی (تند انقباض) موش‌های نر نژاد ویستار می‌باشد. مواد و روش­ها: در این مطالعه تجربی، 14 موش نر ويستار (با ميانگين وزن 15±220 گرم( به‌طور تصادفي به 2 گروه كنترل (7=n) و تمرين مقاومتي (7=n) تقسيم شدند و 12 هفته تمرین را طبق پروتکل اجرا کردند. حیوانات گروه تمرین مقاومتی در قفس فلزي با تور سيمي که 2 بطري آب در بالاترين ارتفاع (2 متری) آن قرار داده مي‌شد، نگهداري شدند. 48 ساعت بعد از آخرين جلسه تمريني، حيوانات بيهوش شدند و عضله نعلی (کند انقباض) و درشت‌نئی قدامی (تند انقباض) آن‌ها جدا شد. برای سنجش nAChR از کیت الایزا استفاده شد. برای آنالیز داده­ها آزمون t مستقل مورد استفاده قرار گرفت.   نتایج: تجزیه‌وتحلیل داده‌ها نشان داد که بین میزان nAChR گروه‌های شاهد و تمرین مقاومتی، در هر دو عضله کند (001/0‌=‌P)‌ و تند (001/0‌=‌P) تفاوت معناداری وجود داشت. بنابراین، تمرین مقاومتی میزان nAChR را در هر دو عضله تند و کند افزایش می‌دهد. همچنین نتايج در گروه تمرین مقاومتی نشان داد كه بين میزان nAChR عضلات کند و تند تفاوت معنادار وجود ندارد (05/0‌>‌P).نتیجه­گیری: میزان  nAChRبا پروتکل تمرین مقاومتی در هر دو عضله تند و کند انقباض، افزایش یافت. به‌نظر‌می‌رسد که تمرين مقاومتي می‌تواند با تسریع عوامل نروتروفیکی (مانند CGRP) که در ساخت و دسته‌بندی nAChR دخیل مي‌باشند، نقش كليدي در افزايش ميزان nAChR داشته باشد

Title: تأثیر فعالیت ورزشی کوتاه و طولانی‌مدت بر شاخص عملکرد سلول‌های بتا در بیماران دیابتی

http://knh.shmu.ac.ir/index.php/site/article/view/125

مقدمه: عوامل مؤثر در بروز دیابت و ايجاد ناتوانی سلول‌های بتا هنوز کاملاً شناخته نشده‌اند. در این مطالعه، اثر فعالیت ورزشی کوتاه و طولانی‌مدت بر عملکرد سلول‌های بتا و حساسیت انسولین در بیماران دیابتی مورد بررسي شده‌است. مواد و روش‌ها: در اين مطالعه مداخله‌اي 30 مرد بزرگ‌سال مبتلا به دیابت نوع 2 با میانگین سنی 43 سال به‌شیوه تصادفی (بلوکی) در 2 گروه تجربی (ورزش) و شاهد (بی‌تمرینی) قرار گرفتند. غلظت گلوکز، حساسیت انسولین و عملکرد سلول‌های بتا در شرایط ناشتا، بلافاصله پس از یک جلسه ورزش زیربیشینه و متعاقب 3 ماه فعالیت ورزشی در گروه تجربی اندازه‌گیری شد. همچنین متغیرهای مذکور در زمان‌های مشابه در گروه شاهد اندازه‌گیری شدند. شاخص‌های آنتروپومتریکی دو گروه، قبل و بعد از ورزشی طولانی‌مدت اندازه‌گیری شدند. از آزمون آناليز واريانس تكراري جهت تجزيه‌وتحليل داد‌ه‌ها استفاده شد. نتایج: کاهش معناداری در گلوکز خون و افزایش معناداری در عملکرد سلول‌های بتا در پاسخ به فعالیت ورزشی کوتاه و طولانی‌مدت در گروه تجربی مشاهده شد (05/0P<). همچنین، حساسیت انسولین در پاسخ به فعالیت ورزشي کوتاه‌مدت، تغییر معناداری پیدا نکرد (05/0P≥)، اما پس از ورزشی طولانی‌مدت افزایش معناداری یافت (05/0P<). در گروه شاهد متغير‌هاي تحت مطالعه تغيير معناداري نداشتند. نتیجه‌گیری: یافته‌های مطالعه ما نشان می‌دهد که کاهش در غلظت گلوکز خون پس از فعالیت ورزشي در بیماران دیابتی به‌دلیل بهبود حساسیت انسولین و عملکرد سلول‌های بتا در این بیماران می‌باشد.

Title: بررسی رابطه هوش هیجانی و موفقیت مدیران آموزشی دانشگاه علوم پزشکی گلستان

http://knh.shmu.ac.ir/index.php/site/article/view/127

مقدمه: هوش هيجاني جديدترين تحول درزمينه ارتباط ميان تفكر و هيجان است و تأثيرات به‌سزايي در موفقيت دارد. هدف از انجام این مطالعه، بررسی ارتباط بین هوش هيجاني و موفقيت مديران آموزشی می‌باشد. مواد و روش­ها: جامعه آماری در اين تحقيق كليه مدیران آموزشی دانشگاه علوم پزشکی گلستان به تعداد 96 نفر (45 نفر زن و 51 نفر مرد) بوده است که به‌ روش سرشماری اطلاعات در سال 1387 جمع‌آوري شد. ابزار جمع‌آوری اطلاعات، پرسش‌نامه‌های دموگرافیک، هوش هیجانی شرينگ و موفقیت بود. رابطه بين میزان هوش هیجانی و میزان موفقیت با استفاده از آزمون ضریب همبستگی اسپیرمن بررسي شد. نتایج: نتایج نشان داد میانگین هوش هیجانی مدیران موفق 7/98 و میانگین هوش هیجانی مدیران ناموفق 7/76 بوده است. ضریب همبستگی اسپیرمن بین هوش هیجانی و موفقیت مدیران 71/0 بوده است که بیان‌کننده رابطه مستقیم بین هوش هیجانی و موفقیت می‌باشد (001/0>P). نتیجه‌گیری: باتوجه به نتایج حاصل از این تحقیق مبنی‌بر وجود رابطه مستقیم بین هوش هیجانی مدیران و موفقیت آنان، به برنامه‌ریزان پیشنهاد می‌گردد به‌منظور بهره‌گیری بیشتر از توان مدیریتی مدیران سازمان‌‌ها، نسبت‌به برگزاری کارگاه‌هایی در جهت افزایش هوش هیجانی آنان، اقدام کنند.

Title: بررسي آگاهي و نگرش دانشجويان مامايي و ماماها درزمینه مامايي جامعه‌نگر

http://knh.shmu.ac.ir/index.php/site/article/view/129

مقدمه: بر‌اساس مطالعات انجام‌شده، مامايي جامعه‌نگر نقش مهمي در ارتقاي سلامت زنان ايفا مي‌كند. هدف از پژوهش حاضر، سنجش آگاهی و نگرش درزمينه مامایی جامعه‌نگر دانشجویان مامایی و ماماهای دانشگاه علوم پزشکی شاهرود می‌باشد. مواد و روش­ها: در این مطالعه مقطعي، پرسش‌نامه‌ای خودساخته مشتمل بر 20 سؤال آگاهی و 25 سؤال نگرش تهیه شده است براي تکمیل در اختیار 102 دانشجوی مامایی و مامای شاغل قرار گرفت که 90 نفر به سؤالات پاسخ دادند. امتیاز آگاهی نهایی به 3 گروه آگاهی ضعیف (امتیاز کمتر از 75)، متوسط (75- 95) و بالا (بالاتر از 95) تقسیم گردید. امتیاز بخش نگرش نیز به 3 قسمت نگرش منفی (25-55)، خنثی (56-85) و مثبت (بالاتر از 85) تقسیم گردید. روایی محتوی پرسش‌نامه طبق نظر اساتید صاحب‌نظر و پایایی آن از‌طریق بازآزمایی مورد تأیید قرار گرفت. نتایج: میانگین نمره آگاهي 1/12±8/89 و میانگین امتياز نگرش در جامعه تحت مطالعه 4/10±8/95 گزارش شده است. بین نمره آگاهی و نگرش دانشجویان مامایی و ماماهای شاغل اختلاف معناداری مشاهده نگردید (05/0(P>. نمره آگاهي در دانشجويان پيوسته به‌صورت معناداري بيش از دانشجويان ناپيوسته بود (05/0(P<. نتیجه­گیری: باتوجه به یافته‌های تحقیق، به‌نظر‌می‌رسد نگرش مثبت نسبت‌به مامایی جامعه‌نگر نشانه وجود زمینه مساعد برای اجرای برنامه‌ها و طرح‌های مداخله‌ای درخصوص مامایی جامعه‌نگر باشد از سوی دیگر باتوجه به آگاهی متوسط درزمينه مامایی جامعه‌نگر توصیه می‌شود، برنامه‌ریزی جهت آموزش بیشتر و تخصصی در‌این‌زمینه انجام گیرد.

Title: کاهش حجم و شیرابه زباله ازطريق طراحی و ساخت دستگاه ابتکاری فشرده‌ساز مکانیکی زباله خانگی

http://knh.shmu.ac.ir/index.php/site/article/view/130

مقدمه: حجم زباله منجر به افزایش هزینه‌های جمع‌آوری، حمل و دفع نهایی آن می‌شود. درصد بالای رطوبت نیز باعث تخریب سریع ماشین‌آلات جمع‌آوری و حمل و افزایش تولید شیرابه در اماکن دفن و درنتیجه افزایش احتمال آلودگی خاک و منابع آب سطحی و زیرزمینی می‌شود، لذا تلاش‌های فراوانی در جهت پیداکردن را‌ه‌حل‌هایی برای مقابله با این دو مشکل در حال انجام است. هدف اين مطالعه، طراحي و ساخت سطل زباله‌اي است كه شيرابه را از تركيب زباله خارج مي‌سازد، درنتيجه حجم خشك زباله كاهش و مقدار شيرابه آن نيز به حداقل مي‌رسد. مواد و روش‌ها: در این مطالعه، به‌دنبال ساخت دستگاه ابتکاری فشرده‌ساز مکانیکی زباله خانگی، اثر آن در کاهش حجم و شیرابه زباله بررسي شد. این دستگاه برای اولین بار در ایران و در دانشگاه علوم پزشکی شاهرود و در مدت 6 ماه طراحی و ساخته شده است‌. به‌منظور تأیید توانمندی‌های این فشرده‌ساز در کاهش حجم و درصد رطوبت زباله، آزمایش‌هايی (3 بار) با استفاده از زباله‌های خشک و تر انجام شد. برای این منظور پس از پرکردن مخزن با زباله تر به‌میزان 18 لیتر و به‌کارانداختن اهرم انتقال نیرو (برای حرکت‌دادن صفحه فشرده‌ساز) توسط فردي 30 ساله با وزن 60 کیلوگرم، حجم و درصد رطوبت، اندازه‌گیری و با مشخصات حجمی و وزنی اولیه زباله مقایسه شد. نتایج: این دستگاه قادر به کاهش قابل ملاحظه حجم زباله و جداسازی رطوبت زباله‌های تر به‌ميزان 45% مقدار اولیه (به‌طور ميانگين) است و تکرار آزمایش با زباله‌های خشک نیز حاکی از توانایی این سطل برای کاهش حجم زباله خشک به‌ميزان 40% حجم اولیه آن (به‌طور ميانگين) بود‌. نتیجه‌گیری: اين دستگاه مي‌تواند حجم زباله را به‌میزان قابل توجهي کاهش دهد و شيرابه را نیز از تركيب زباله خانگي استخراج و جداسازي نمايد و درنتيجه پیامدهاي منفي زيست‌محيطي زباله و مشكلات جمع‌آوري آن را كاهش دهد.

Title: گزارش یک مورد درمان زخم پای دیابتی با استفاده از بره موم حرارت‌دیده در داخل روغن زیتون

http://knh.shmu.ac.ir/index.php/site/article/view/132

مقدمه: زخم پای دیابتی، یکی از مشکلات اصلی سلامتی در افراد دیابتی می‌باشد. امروزه چندین روش برای درمان زخم پای دیابتی وجود دارد، ولی با‌این‌حال بعضی از بیماران ‌مجبور به ‌قطع‌ عضو می‌شوند. زن 45 ساله‌اي كه سابقه‌ دیابت نوع ‌دو داشت، با زخمی‌ در ناحیه‌ انگشت بزرگ پا که به‌علت عدم‌رعایت بهداشت پا 3 ماه قبل ایجاد شده بود، به کلینیک دیابت و غدد مراجعه کرد. زخم در این مدت با پودر پنی‌سیلین و بتادین تحت درمان قرار گرفته، ولی هیچ‌گونه بهبودی حاصل نشده بود و به‌علت‌ عفونت شدید بیمار، جراح به قطع عضو او توصیه كرده بود. در این گزارش نمونه‌ای از درمان زخم پای دیابتی با بره موم حرارت‌دیده در داخل روغن زیتون معرفی می‌گردد. مواد و روش‌ها: بیمار هنگام مراجعه زخمی در ناحیه انگشت پا به ابعاد 2×1 سانتی‌متر مربع و عمق 1 سانتی‌متر داشت. بعد از مراجعه، استفاده از پودر پنی‌سیلین و شستشو با بتادین قطع و درمان به‌صورت پانسمان روزانه با مخلوط موضعی از بره موم حرارت‌دیده در داخل روغن زیتون و روغن حیوانی شروع شد. به بیمار توصیه شد که زخم خود را قبل از پانسمان با سرم شستشو دهد و سپس مخلوط موضعی را روی یک گاز استریل به ابعاد 4×4 بگذارد و روی محل زخم قرار دهد و پانسمان هر 12 ساعت یک‌بار عوض شود. نتایج: بعد از گذشت یک هفته از شروع درمان، بافت جدید در زخم دیده شد و زخم در طول 2 هفته کاملاً بسته شد. نتیجه‌گیری:. بره موم حرارت‌دیده در داخل روغن زیتون و روغن حیوانی برای درمان زخم پای دیابتی مناسب است.
